# Random Amino Acid Mutations and Protein Misfolding Lead to Shannon Limit in Sequence-Structure Communication

**DOI:** 10.1371/journal.pone.0003110

**Published:** 2008-09-01

**Authors:** Andreas Martin Lisewski

**Affiliations:** Department of Molecular and Human Genetics, Baylor College of Medicine, Houston, Texas, United States of America; University College London, United Kingdom

## Abstract

The transmission of genomic information from coding sequence to protein structure during protein synthesis is subject to stochastic errors. To analyze transmission limits in the presence of spurious errors, Shannon's noisy channel theorem is applied to a communication channel between amino acid sequences and their structures established from a large-scale statistical analysis of protein atomic coordinates. While Shannon's theorem confirms that in close to native conformations information is transmitted with limited error probability, additional random errors in sequence (amino acid substitutions) and in structure (structural defects) trigger a decrease in communication capacity toward a Shannon limit at 0.010 bits per amino acid symbol at which communication breaks down. In several controls, simulated error rates above a critical threshold and models of unfolded structures always produce capacities below this limiting value. Thus an essential biological system can be realistically modeled as a digital communication channel that is (a) sensitive to random errors and (b) restricted by a Shannon error limit. This forms a novel basis for predictions consistent with observed rates of defective ribosomal products during protein synthesis, and with the estimated excess of mutual information in protein contact potentials.

## Introduction

In the sixty years since its formulation communication theory [1] has shaped modern technology, from integrated circuits to satellite communication. Claude Shannon's fundamental insight was that, with the right code, information can be reliably transmitted between sender and receiver at any level of spurious noise, although the practical design or discovery of such Shannon codes has proved challenging.

The generality of Shannon's results suggests that biological systems may also use Shannon codes, such as in the transfer of genomic information during cellular protein synthesis. Despite efforts over the last fifty years [2], evidence for this hypothesis has remained inconclusive [3], [5]. Yockey, who pioneered an information theory approach to the Central Dogma [6], applied the Shannon-Weaver communication model [1] to describe the flow of information from DNA to the amino acid sequence but did not provide a detailed information theoretic description of the folded state. Entropy analysis may indicate that the ‘information content’ of the physical protein structure is large enough to accommodate the ∼4 bits per amino acid residue in primary sequence [7], [8]. However, ∼4 bits per residue cannot be the true rate of information transfer between sequence and structure. This follows from (a) Anfinsen's result that a fully translated amino acid sequence is necessary and sufficient for a protein to fold into its native state [9], and from (b) Levinthal's argument that folding cannot be realistically achieved by sampling an astronomical number of configurations [10]. In contradiction to (b), such a high rate would require, for a typical protein of ∼400 amino acids, any receiver to decode the correct state from ∼2^1600^ possible states. Furthermore, given (a), there is no way to avoid this combinatorial explosion by determining the correct protein shape from a lesser part of the amino acid sequence. Thus, for information transmission between sequence and structure to be realistic, transmission rate must be much smaller than ∼4 bits per residue.

In line with this argument, mutual information studies show that information exchange between primary and secondary structure is ∼0.20 bits per amino acid residue [11], which is a factor five higher than estimates between primary and tertiary structure in contacts of native structures [12], [13]. Because non-local contacts mainly determine tertiary structure, this implies that information transfer between sequence and tertiary structure is indeed modest, a few hundredth of a bit per residue [11]–[13].

The main result in information theory is Shannon's noisy channel theorem which sets a universal limit on communication in any error prone communication channel [1]: the Shannon limit. It says that communication can take place only if channel capacity *C* is above the transmission rate *R*. Although no reliable communication in Shannon's sense is possible below this point a Shannon limit has not been explicitly proposed as part of a *communication protocol* between sequence and structure.

This situation appears unsatisfactory given the growing evidence that error in protein synthesis is common: ∼30% of all ribosomal products in eukaryotic cells are degraded during or immediately after translation and folding suggesting that a large fraction of proteins is synthesized into aberrant structures (misfolded protein) [14], [15]. This is significantly higher than the error accumulated during translation, which amounts to 4×10^−4^ per residue [16], and therefore corresponds, for an average chain length of ∼400, to only ∼0.2 amino acid errors per completed protein chain. Furthermore, misfolded proteins appear to play critical roles in prevalent diseases such as Alzheimer's, Parkinson's or diabetes [17]–[19]. Hence, an adequate model of cellular protein synthesis should address errors explicitly.

Here, to support the hypothesis that a noisy communication channel with a Shannon limit exists in the protein sequence-structure map, we encode a large set of experimental protein atomic coordinates into a contact vector representation [20]. This discrete and one-dimensional representation of tertiary structure, which orders all polypeptide backbone hydrogen bonds by their sequence separation, leads to two main results. First, it gives quantitative evidence for a communication channel with an information capacity *C* above a Shannon limit at 10^−2^ bits per amino acid symbol. Second, it introduces a measure of communication fidelity between sequence and structure, the Gallager probability of error-free communication *q_e_^−^*. Above the Shannon limit both measures are sensitive to errors in crystallographic structures and in primary sequence. By contrast, models of misfolded structures and random coils do not achieve the Shannon limit, i.e. capacity falls below 10^−2^ bits per amino acid symbol and communication fidelity vanishes exactly.

These results are consistent with studies on the efficacy of protein synthesis and sequence-structure correlation, including (a) the high rate (∼30%) of ‘defective ribosomal products’ in eukaryotic cells [14], [15], which equals the error probability derived from high-resolution protein structures, (b) mutual information estimates between sequence and structure [11]–[13], which are consistent with channel capacities given here, and (c) the observed excess in mutual information from protein contact potentials [12], which matches the reported Shannon limit.

We conclude that the sequence-structure map in proteins can be represented in a biologically meaningful way as a noisy digital communication channel with an output error probability of at least ∼30% and a Shannon limit at 10^−2^ bits per amino acid symbol.

## Materials and Methods

### Model formulation: Shannon-Weaver communication between protein sequences and structures

**Figure 1:**
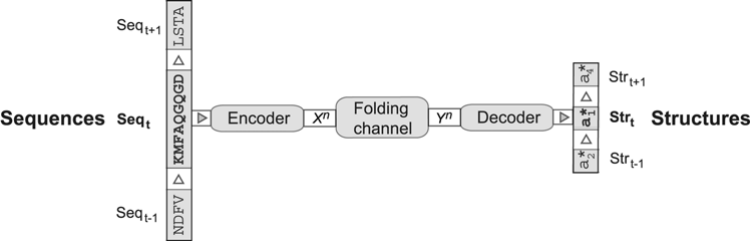
Shannon-Weaver communication model of serial protein synthesis. A series of amino acid sequences {…, Seq*_t_*_−1_, Seq*_t_*, Seq*_t_*_+1_} = {…, NDFV, KMFAQGQGD, LSTA, …} is encoded, one sequence at a time into one code word *X^n^*, transmitted over the folding channel to an output code word *Y^n^*, and finally decoded into structural symbols {…, a*_2_, a*_1_, a*_4_,…} which represent the folded structures {…, Str*_t_*_−1_, Str*_t_*, Str*_t_*_+1_}.

Cellular production of polypeptides was modeled as a serial process where over time many chains are synthesized by the translational and ribosomal apparatus. Figure 1 shows a schematic: translation determines a series of amino acid sequences {…, Seq*_t_*_−1_, Seq*_t_*, Seq*_t_*_+1_, …} = {Seq*_t_*}*_t_*_∈**Z**_, each Seq*_t_* for one protein chain, ordered by a discrete temporal order *t*∈**Z** of corresponding tertiary structures {…, Str*_t_*_−1_, Str*_t_*, Str*_t_*_+1_,…} = {Str*_t_*}*_t_*_∈**Z**_, where **Z** = {…,−1, 0, 1,…} is the set of integers. For example, translation and folding of sequence Seq*_t_*_−1_ into a structure Str*_t_*_−1_ was completed before it was finished so for Seq*_t_*. Thus the synthesis of individual polypeptide chains is ordered by a discrete time index representing source and destination random processes {Seq*_t_*}*_t_*_∈**Z**_ and {Str*_t_*}*_t_*_∈**Z**_, respectively.

Our model hypothesis was a Shannon-Weaver communication channel [1] between amino acid sequences (the source, or sender) and corresponding structures (the destination, or receiver). Source and destination are linked with three consecutive components: an encoder, a noisy channel, and a decoder.

The source is here defined as a series of concatenated primary sequences {Seq*_t_*}*_t_*_∈**Z**_ resulting in a stream *S_A_* of letters from the amino acid alphabet *A* with alphabet size |*A*| = 20. The encoder is a map that uses a block code of fixed length *n* to encode the source through a set of code words (the code book), i.e., it maps every sequence Seq*_t_* onto one single code word *X^n^*(Seq*_t_*) represented by an *n*-vector (*x*_1_,…, *x_n_*) of integers. The code word is an element of the code book A^*^, the finite set of all code words. The message input *X^n^*(Seq*_t_*) = (*x_1_*,…, *x_n_*) is transmitted over a noisy communication channel which outputs an *n*-vector *Y^n^*(Str*_t_*) = (*y_1_*,…, *y_n_*), now representing the folded protein chain Str*_t_*. This step mirrors the physical folding process in which a geometrically unspecified sequence becomes a functionally determined 3D structure, and communicational noise is interpreted as any physical interaction of the nascent protein with its environment so that the original input *X^n^* is randomly distorted into an output *Y^n^*. In a last step, a decoder deciphers *Y^n^*(Str*_t_*) by selecting one member in the code book A^*^ that registers the completed structure. This decoding produces an output sequence S_A*_ of structural symbols in A^*^ and it completes the communication process. These communication channel components were established from structural protein data as follows.

### Protein structural data sets

The representative set of *N_P_* = 31609 protein tertiary structures and their primary sequences was taken from the Research Collaboration for Structural Bioinformatics Protein Data Bank (PDB) [21] in September 2005. Redundancy was limited only to the extent that multiple chains with identical sequences from the same PDB file were removed, and the complete list of PDB chain identifiers was deposited at http://mammoth.bcm.tmc.edu/lisewski2008/np.list. A smaller and non-redundant subset of *N*_25_ = 2372 protein chains represented the PDBselect25 list [22] from March 2006.

### Misfolded protein structures data set

The library of 928 chains and their misfolded C_α_ backbone coordinates in PDB file format was deposited at http://mammoth.bcm.tmc.edu/lisewski2008/misfold928.tar.gz as a compressed UNIX tar-archive.

### Channel output and input

**Figure 2:**
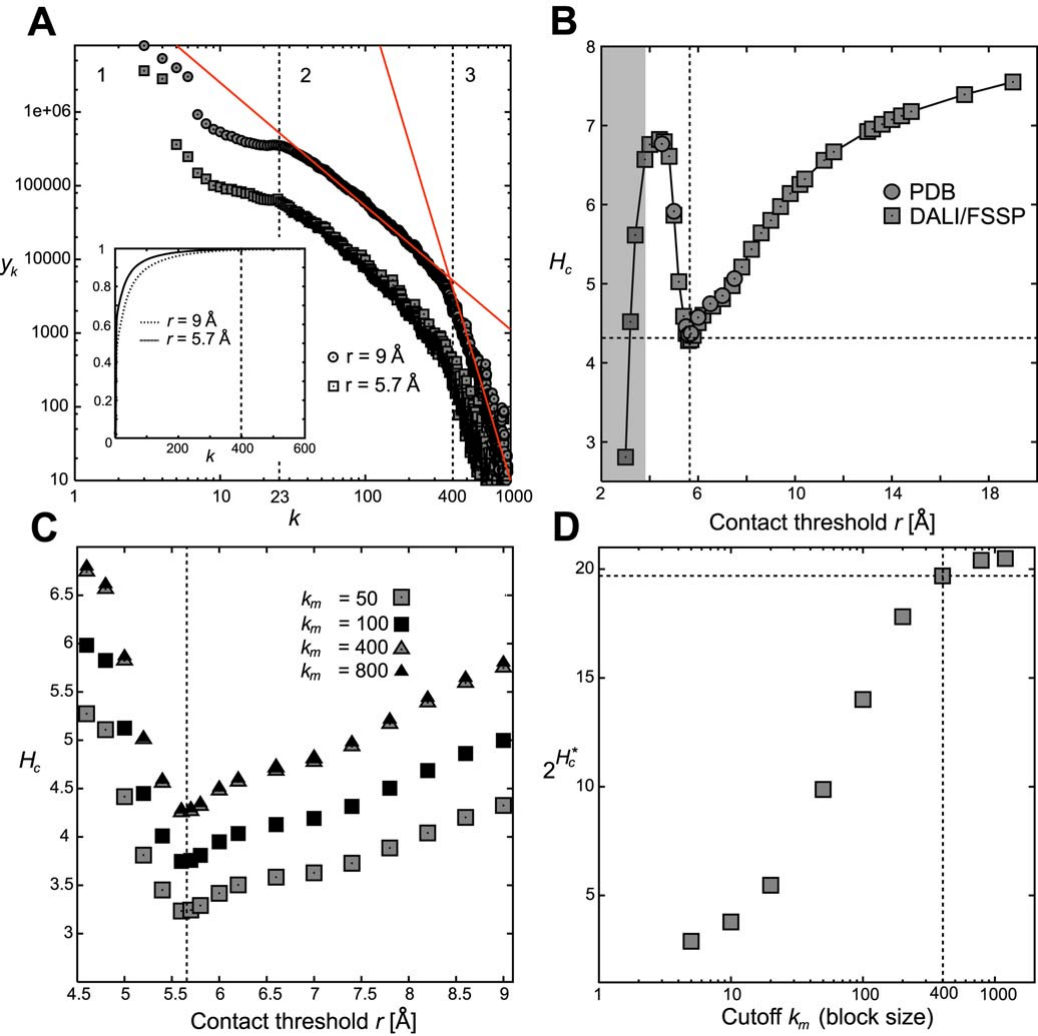
Block code of contact vectors represents protein tertiary structures. (A) Absolute distributions of contacts ordered by their contact lengths *k* for 31609 structures from the Protein Data Bank evaluated at two choices of the contact threshold *r*. Contributions for *k*>400 are negligible; insert shows the corresponding cumulative distributions. Solid red lines indicate linear fits to range (2) and range (3). (B) Information (Shannon) entropy *H_c_* of contact vectors across different choices of contact thresholds *r* and for two collections of PDB structures (‘PDB’, a set of 31609 PDB chains; ‘DALI/FSSP’, a set of 3000 structural domains). Grey region indicates thresholds *r* below the distance of two consecutive C_α_ atoms in the polypeptide chain. (C) Contact vector information entropy *H_c_* as a function of contact threshold *r* and contact vector cut-off *k_m_*. Dashed vertical line depicts the minimum at *r_m_* = 5.7 Å. (D) Rise and saturation at ∼log_2_ 20 of the minimum entropy H_c_^*^ with increasing contact vector length *k_m_*. A choice of *k_m_* = 400 was sufficient to reach the asymptotic value (dashed lines).

For the channel output we have chosen a unique one-dimensional contact vector representation of the folded polypeptide chain [20]. A contact vector is the integer-valued distribution *y_k_* counting at each component all contacts that are separated by *k*−1 steps along the sequence, with *k*≥3 (residue pairs with *k*<3 are always in contact). Since chains vary in length, the maximum value of *k* for which *y_k_* does not vanish depends on the given structure. A large-scale analysis showed that there exists a natural cut-off for *k*, and contacts with longer sequence separations contributed significantly less [23]. To verify this, we calculated the absolute distribution from *N_P_* = 31609 PDB chains for two choices, 5.7Å and 9Å, of the geometrical distance threshold *r* which defines a contact pair if any two C_α_ atoms of the backbone are closer than *r* (Fig. 2A). For *k*>400 the distribution rapidly dropped with a negative slope of *m*≈−4.7 (double-log scale), and the cumulative distribution indicated that relative contributions above *k_m_*≈400 were negligible (insert in Fig. 2A). This behavior was not sensitive to a particular choice of *r*, provided *r* was larger than the distance between consecutive C_α_ atoms (Fig. 2A). Therefore every channel output *Y^n^*(*Str_t_*) defined a contact vector (*y_3_*,…, *y_n+2_*) with block length *n* = 400.

To control how channel output depended on the geometrical contact distance *r*, we normalized the distribution of *y_k_* and calculated its Shannon entropy *H_c_* = −Σ*_k_ y_k_* log_2_ *y_k_*. The entropy *H_c_*(*r*) was traced over increasing *r*, from *r* = 3.8 Å onward, and we observed a unique minimum at *r_m_* = 5.7Å with *H_c_* ^*^ = 4.28≈log_2_ 20 bits (Fig. 2B). This minimum was the same for two different choices of native protein structures, the whole set of *N_p_* = 31609 PDB structures and a non-redundant subset of 3000 single domain chains from the DALI/FSSP database [24], and it therefore was independent of the number of domains per chain. Also, the minimum did not depend on the block length if *k_m_*>400 (Fig. 2B).

This observation implied that an alphabet of no more than 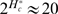 symbols (size of the code book) was necessary to represent contact vectors with minimum redundancy. Thus, to minimize redundancy, we fixed *r* = *r_m_* as a geometric contact threshold between residues. This step was equivalent to taking the least cost *t_k_*∼−log_2_(*y_k_*) for decoding [25], [26] by minimizing the entropy Σ*_k_ y_k_ t_k_* = *H_c_*(*r*).

The chosen contact threshold equals the average distance between two C_α_ carbons in backbone hydrogen bonds at 5.77±0.53 Å. Hydrogen bonds were identified from a given atomic record using the Hydrogen Bond Explorer computer program version 2.01 with default parameter settings [27]. Hence contact vectors have a distinct biophysical meaning: they estimate the number of backbone hydrogen bonds ordered by sequence separation.

With these choices, block length *n* = 400 and contact threshold *r_m_* = 5.7Å, we characterized the block code of contact vectors and no further parameters were included in our model.

### Decoder and code book

**Figure 3:**
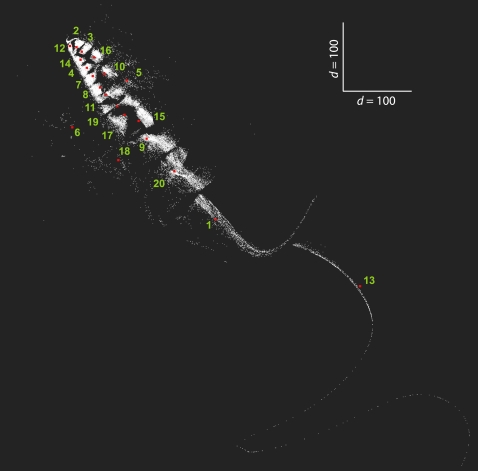
Map of contact vectors from the Protein Data Bank. Multidimensional scaling 2D map of 31609 contact vectors extracted from the Protein Data Bank. Red dots indicate the position of the code words in A*, which represent twenty clusters in contact vector space as listed in Table S1 (Supporting Information). Shorter chains are in the upper left corner while longer chains are located in the lower right corner of the map.

For decoding a set A^*^ of code words (the code book) was specified through a cluster detection method among all contact vectors. Since for our data an optimum code book was estimated to have 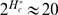 code words, we used a standard heuristic and applied the *k*-means algorithm with *k* = 20 over the space of *N_P_* = 31609 contact vectors to identify the elements in A^*^. Cluster algorithms like *k*-means approximate a given set of many feature vectors by a much smaller number of representative vectors [28]. Algorithmic convergence was reached rapidly and resulted in a set of twenty code words A^*^ = {*a* ^*^_1_, …, *a* ^*^_20_}, where each *a* ^*^_i_∈A^*^ was a single contact vector. Figure 3 shows these twenty code words (red dots) embedded among all *N_P_* contact vectors in a reduced two-dimensional map projected with multidimensional scaling (MDS).

Following standard practice, decoding was done through vector quantization [28]: any channel output *Y^n^*(Str*_t_*) was assigned to the nearest codeword *a* ^*^ *_min_*∈A^*^ according to the nearest neighbor condition
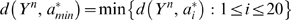
with the contact metric distance [21], *d*(*X^n^*, *Y^n^*) = Σ*_k_* |*x_k_*−*y_k_*|.

### Source and destination

**Figure 4:**
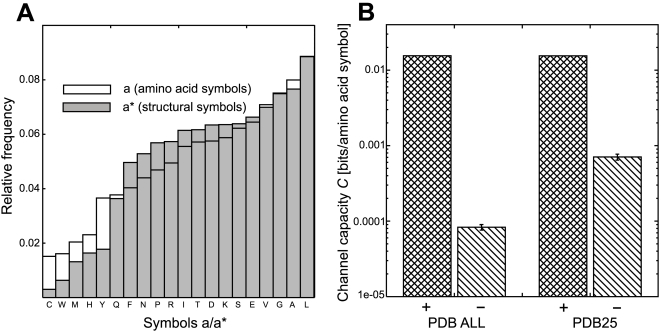
Statistical distributions of amino acid symbols and structural symbols. (A) Relative frequencies of amino acid symbols (*a*∈*A*) and structural letters (a^*^∈A^*^) from the set of 31609 chains in the Protein Data Bank. Both symbol alphabets have similar information entropies: *H*(*A*) = 3.90 bits and *H*(A^*^) = 3.76 bits. (B) Estimates (+) on the channel capacity *C* for two sets of structures (‘PDB ALL’, a collection of 31609 PDB chains; ‘PDB25’, a subset of 2372 proteins with low sequence redundancy.) Negative controls (−) after 100 random permutations of the sequences *S_A_* and S_A*_ for both sets (bars indicate standard deviations).

Source and destination were two symbol sequences, *S_A_* and *S_A_* ^*^, at each side of the communication channel: one sequence of |*S_A_*| = 7702314 amino acid symbols and a second sequence of |*S_A_* ^*^| = 31609 corresponding structural symbols in *A* ^*^. Statistically, both sequences had similar symbol distributions (Fig. 4A) with Shannon entropy *H*(*A*) = 3.90 bits for the amino acid alphabet *A*, and *H*(*A* ^*^) = 3.76 bits for structural code words in *A* ^*^. Finite sampling effects underestimate the Shannon entropy by *M*/2*N*, where *M* is the number of symbols in the sample (here, *M* = 20), and *N* = |*S_A_*|+|*S_A_* ^*^| is the sample size [29]. This yielded negligible corrections of 3×10^−4^ for *H*(*A* ^*^) and 1×10^−6^ for *H*(*A*).

A control showed that a single code word in *A* ^*^ with *H_A_* ^*^ = 3.76 bits of information was sufficient to identify the native conformation among all known protein structures. This was consistent with two observations: (1) every contact vector trivially determines the amino acid sequence length (consecutive residues are always in contact in the polypeptide chain), (2) given a single domain chain, only *H*_CA_ = 3.54≈*H_A_* ^*^ bits of information are necessary to determine the structural class and architecture, i.e. the first two levels in the CATH hierarchy (the “Class Architecture Topology Homologous superfamily” classification of protein structure domains, version 2.6.0, [30]). *H*_CA_ is the information entropy from the distribution of known structural domains among all 39 protein architectures of CATH version 2.6.0.

We further tested if sequence length and domain architecture were sufficient to identify the correct fold. The test set were 5160 single domain structures with known CATH architecture from the set of *N_P_* = 31609. For every chain in the test set the most similar other was chosen from the entire pool of *N_P_* with the smallest difference in sequence length among those sharing the same domain architecture in CATH. All identified pairs were then geometrically aligned with the FAST algorithm (Fast Alignment and Search Tool, [31]) yielding an all-atom alignment RMSD of 2.54±2.23 Å with a fraction of 0.63±0.32 of the residues aligned. Thus, on average, both chains were representatives of the same fold by common criteria [32].

### Channel capacity, rate and Shannon's theorem

The channel capacity was numerically derived in two steps. First, the conditional probability *p*(*A*|A^*^) was defined as an event counting table, where rows represent the 20 possible structural symbols {*a*_1_^*^, *a*_2_^*^, …}, and columns the 20 amino acids symbols {A, G, …}. Thus, for a source amino acid sequence and a single destination symbol the entries in a given row were incremented accordingly. This was done for all *N_P_* = 31,609 protein chains, and the table was normalized such that Σ*_a_*_∈*A*_ *p*(*a*, *a* ^*^) = 1 for all *a* ^*^∈*A* ^*^. Second, the joint probability is *p*(*A*, *A* ^*^) = *p*(*A*|*A* ^*^) *p*(*A* ^*^), and from it the mutual information can be calculated as *I*(*A*; *A* ^*^) = −Σ*_a_*_∈*A*_ Σ*_a_*_∈A*_ *p*(*a*, *a* ^*^) log_2_ *p*(*a*, *a* ^*^)/(*p*(*a*) *p*(*a* ^*^)). The full 20×20 table *p*(*A*, *A* ^*^) was deposited at http://mammoth.bcm.tmc.edu/lisewski2008/np31609_joinp.dat.

The channel capacity *C* is defined as
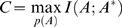
In this formula, the maximum in mutual information over distributions *p*(*A*) was achieved through the symbol frequencies in *S_A_*, since they represent the fixed natural amino acid propensities in biological organisms. The channel capacity gives the maximum amount of information that can be transferred in a single use of the channel. A *single use of the channel* is the transmission of a single amino acid symbol.

The code rate *R* is defined as *R* = *H*(*A*)/*n*, where *H*(*A*) is the information entropy of the amino acid sequence (source) and *n* is the code block length used by the encoder.

If code rate *R* and channel capacity *C* are known, then Shannon's theorem tells us whether communication over the channel is possible. The case *C*>*R* implies that for every block size *n*>*n_min_* = *H*(*A*)/*C* Shannon codes exist, whereby information can be transmitted over the communication channel with arbitrary small error, i.e., the probability *p_e_* for a mismatch at the decoder is bounded from below by zero. The opposite case, *C*<*R*, signals a breakdown of reliable transmission: no Shannon code exists and *p_e_* ceases to be bounded from below by zero, thus approaching one exponentially with increasing block length *n*. The point where capacity *C* equals rate *R* is the Shannon limit.

## Results

### Native protein structures satisfy Shannon's theorem

A direct application of Shannon's noisy channel theorem confirmed that communication between protein amino acid sequences and native structures was achievable. The sequence and structural data from the Protein Data Bank (PDB) yielded a Shannon entropy of the amino acid sequences at *H*(*A*) = 3.90 bits and, with the block length *n* = 400, a transmission rate *R* = 0.010 bits per amino acid symbol followed. Notice that the rate does depend on the composition but not on the amino acid order. The estimated channel capacity from these data was *C* = 0.016 bits/amino acid symbol (Fig. 4B). A negative control was done by generating 100 random realizations of *S_A_* and *S_A_* ^*^, in which all symbols in the original sequences were randomly permuted (so that the relative frequencies of symbols were preserved.) This gave an average capacity (8.3±0.7)×10^−5^ bits/amino acid symbol, which is a 180-fold decrease with respect to the positive result (Fig. 4B).

To test whether this capacity estimate was sensitive to sample size and redundancy, we turned to a smaller subset of proteins from the PDB, restricted to *N*_25_ = 2372 protein chains with mutual sequence identity of less than 25%. This choice resulted in a capacity of *C*_25_ = 0.016 bits/amino acid symbol. A negative control through 100 random permutations yielded (7.1±0.064 )×10^−4^<0.05 *C*_25_, see Fig. 4B, and it showed that this estimate was robust.

Thus for the given set of native protein structures channel capacity *C* was 0.006 bits/amino acid symbol above the rate *R* and therefore, as expected, communication from amino acid sequences to tertiary structures was achieved.

### Random errors lead to Shannon limit

To monitor the response of the communication channel to random errors we used channel capacity *C* and the related Gallager error bound *p_e_* ^−^ as indicators [33]. The latter gives an upper limit *p_e_* ^−^ for the decoder error probability *p_e_* of the best possible code with block length *n* (the code with the lowest error probability) [34], viz.
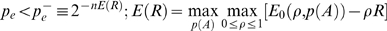

*R* is the rate, *p*(*A*) is the distribution over the amino acid alphabet, and
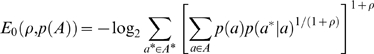
As above for the channel capacity, the maximum over the source's distribution in was given through the natural amino acid frequencies, and *p_e_* ^−^ was computed from the structural data of *N_P_* protein chains. Since *p_e_* ^−^ gives an upper bound for the probability of channel error, we hypothesized that it represents a measure of communication fidelity between protein sequences and structures.

To test this hypothesis, we evaluated the Gallager error bound against random errors imposed onto the symbol sequences *S_A_* and *S_A_* ^*^. If information can be transmitted between the symbol streams *S_A_* and *S_A_* ^*^, then random substitutions on either side of the channel should lower mutual information and therefore also reduce the channel capacity *C*. Concurrently, reducing the capacity to the limit *C* → *R* implies *p^−^_e_* → 1, since no information can be reliably transmitted at capacities less than the rate *R*, according to Shannon's theorem.

**Figure 5:**
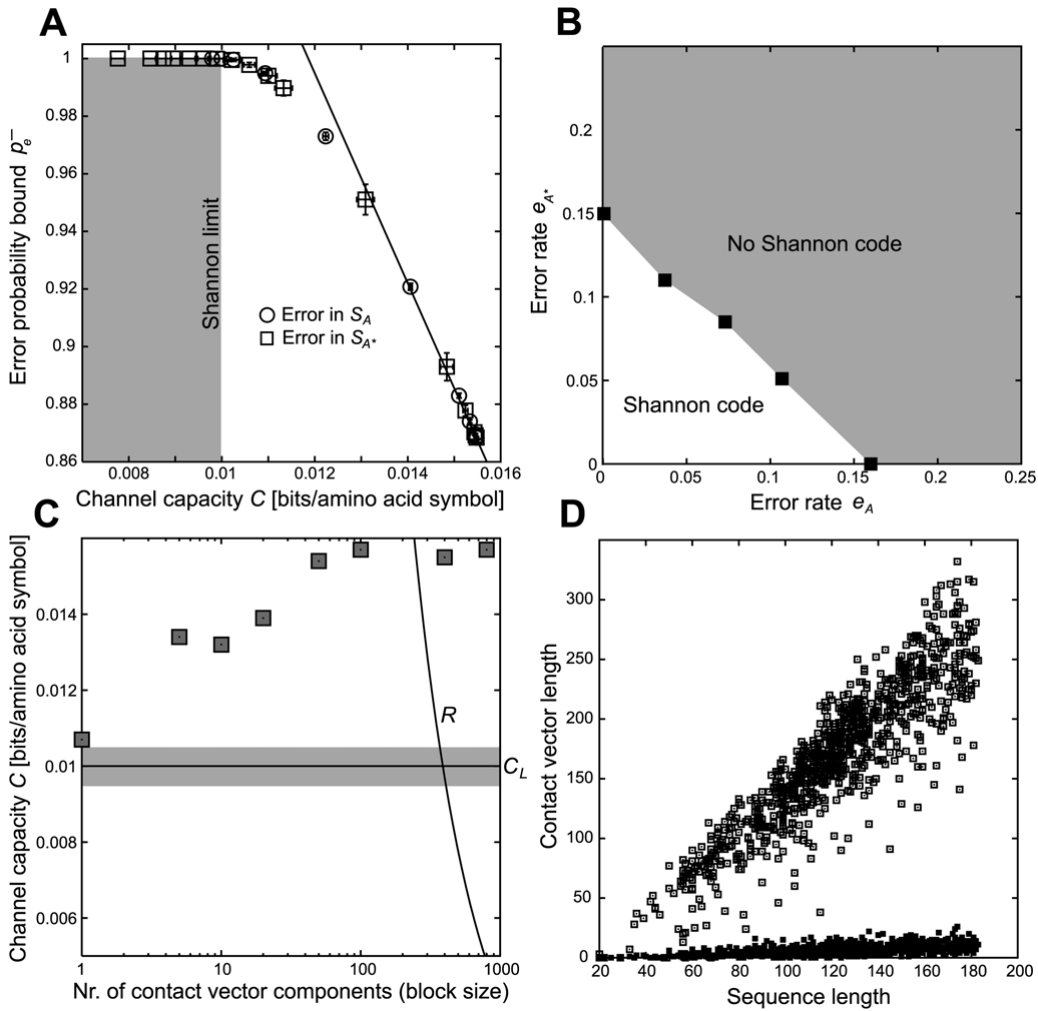
Random errors lead to Shannon limit in sequence-structure communication. (A) The Gallager error bound *p_e_* ^−^ as a function of capacity *C*. Channel capacities were derived at increasing error rates *e_A_* and e_A*_ among the symbols in *S_A_* (circles) and S_A*_ (squares), respectively. At a limit capacity *C* = 0.010 bits/amino acid symbol, *p_e_^−^* becomes one. Bars represent standard deviations over 100 random realizations. (B) Two linearly separated regions with *p_e_^−^*<1 for *e_A_*+e_A*_<*e_max_* (‘Shannon code’), and with *p_e_* ^−^ = 1 for *e_A_*+e_A*_>e_max_ (‘No Shannon code’), with e_max_ = 0.15. The separating line between these regions indicates the Shannon limit. (C) Channel capacity as a function of a contact vectors with a reduced number *n* of components (block size). Capacities *C* above the transmission rate *R* allow communication. *C_L_* is the capacity from a sample of ten random code books representing only chain lengths *L* (grey region depicts standard deviation). (D) Strong correlation between amino acid sequence length and total number of contacts in a contact vector for a control set of 928 structural models of native structures (open boxes); loss of correlation for the same set of unfolded structural models.

In the first test we did not change the elements in *S_A_* ^*^, but imposed errors in *S_A_* through artificial missense mutations by randomly substituting amino acid symbols at increasing rates *e_A_*. We asked to what extent the given structural message *S_A_* ^*^ at destination was compatible with random perturbations in amino acid sequence *S_A_*. For example, *e_A_* = 0.01 meant that one percent randomly selected symbols in *S_A_* were randomly substituted with different amino acid symbols. Twelve error levels *e_A_* between 0 and 0.20 were selected each over an ensemble over 100 random realizations. Figure 5A (circles) shows *p_e_* ^−^ plotted against *C*, where data points at the lower right corner correspond to lowest values of *e_A_*, with a minimum *p_e_*_,0_^−^ = 0.86 for *e*_A_ = 0. As expected, increase in *e*_A_ lead to a drop in *C* and an increase in *p_e_* ^−^. At capacities lower than *C* = 0.010 bits/amino acid symbol, the Gallager bound saturated at maximum value *p_e_* ^−^ = 1 with vanishing standard deviations.

In a second test, to analyze errors at the destination, we left unchanged the amino acid symbols in *S_A_* but this time put errors into *S_A_* ^*^ at increasing rates *e_A_* ^*^. Error rates were selected at sixteen levels between *e_A_* ^*^ = 0 and *e_A_* ^*^ = 0.20. For vanishing *e_A_* ^*^ the lowest value for the Gallager bound again was *p_e_*_,0_^−^ = 0.86. For increasing errors *e_A_* ^*^ (Fig. 5A, squares) channel capacity *C* and error bound *p_e_* ^−^ were along the same curve as in the previous case, and in particular capacities below *C* = 1.0×10^−2^ bits/amino acid symbol implied *p_e_* ^−^ = 1 with vanishing standard deviations. When combined, this equivalent response to errors in *S_A_* and *S_A_* ^*^ indicated a linear relation between *e_A_* and *e_A_* ^*^ at the limit where *p_e_* ^−^ reached its maximum, *e_A_*+*e_A_* ^*^ = *e_max_*, with *e*_max_≈0.15. Therefore errors in the symbol sequences *S_A_* and *S_A_* ^*^ were additive in defining two linearly separated regions: *p_e_* ^−^ = 1 for *e_A_*+*e_A_* ^*^≥*e*_max_ (grey region in Fig. 5B), and *p_e_* ^−^<1 for *e_A_*+*e_A_* ^*^<*e*_max_ (white region in Fig. 5B).

As a main result, the error rate *e*_max_ at which the Gallager bound became maximal, *p_e_* ^−^ = 1, indicated a Shannon limit because at this point the rate *R* = 0.010 bits/amino acid symbol and the capacity *C* were equal (Fig. 5A). This conclusion was further supported by the observation that *e*_max_ and the minimum error bound *p_e_*_,0_^−^ were exhaustive: *e*_max_+*p_e_*_,0_^−^ = 0.15+0.86≈1. Thus randomly adding errors at source or destination up to a limit *e*_max_ maximized the Gallager bound *p_e_* ^−^ and lowered *C* to the level where it equaled *R*. The Gallager error bound *p_e_* ^−^ therefore established a measure of sequence-structure fidelity in proteins, defined as *q_e_^−^* = 1−*p_e_^−^*.

The Shannon limit is the point where contact vectors transmit merely one structural attribute over the communication channel: the protein's chain length *L*. This proposition was in line with two controls. First, ten code books simply defined by twenty random chain lengths yielded a capacity *C_L_* = 0.0100±0.0006 bits per chancel use (Fig. 5C), which indicates that ∼60% of the channel capacity at *C* = 0.016 bits per amino acid symbol could be assigned to a transmission of chain lengths. Second, reducing contact vector length, from *n* = 400 to *n* = 1, lead to a decrease in channel capacity to the point where *C*≈*C_L_*, see Fig. 5C. Since contact vector components in native structures are proportional to sequence length (correlation coefficient *r* = 0.92 with *C* = 0.010 bits per amino acid symbol for a control set of 928 native chains, Figure 5D), the Shannon limit at *C_L_* becomes the least amount of reliable information about the structure that a contact vector may carry.

A negative control confirmed this statement (Figure 5D): for a corresponding set of 928 modeled random coils channel capacity dropped to *C* = 0.003 bits per amino acid symbol and correlation was poor (*r* = 0.53). Thus unfolded chains such as random coils do not achieve the Shannon limit at 0.010 bits per amino acid symbol.

### Errors in tertiary structure impair communication

**Figure 6:**
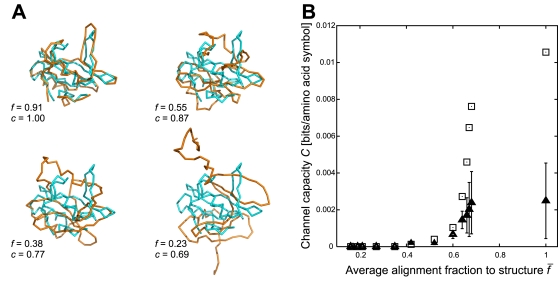
Loss of native geometry in protein structures leads to channel capacities below the Shannon limit. (A) For the PDB structure 1M27 chain A (a phosphotransferase), the four panels show how loss of native contacts (smaller *c*) leads to C_α_ model backbones (magenta backbone trace) with a smaller alignment fraction to the native fold (cyan). (B) Channel capacity *C* for a control set of 928 structures at ten levels of geometrical deformation from the native state as measured by the average alignment fraction (open boxes); negative controls after ten random permutations (triangles with standard deviation error bars).

In order to test how structural deviations from the native state distort communication between sequence and structure, we generated a series of increasingly distorted structures for each one in the control set of 928 native chains. Structural models were C_α_ backbones created through a contact potential Monte Carlo optimization algorithm [35], which recovered a physically realistic C_α_ backbone from the protein's contact map and its primary sequence. To generate misfolded chains, we randomly removed contacts from native contacts maps and used the reduced maps as input for the algorithm. The fraction *c* indicates the remaining contacts in the contact map, such that *c* = 1 corresponds to the native structure. For example, Fig. 6A shows four output models at values *c*∈{1.00, 0.87, 0.77, 0.69} applied to the A chain of PDB entry 1M27, a phosphotransferase with an SH2 domain. The level of deformation from native geometry was measured with the FAST algorithm [31], which for each value *c* calculated a corresponding alignment fraction *f*, defined as the number of aligned residues over the total number of residues. As the example shows, loss of native contacts (smaller *c*) lead to model structures with lesser geometric similarity to the native fold (smaller *f*).

For every chain in the set of 928 a series of ten misfolded models with decreasing values 1≥*c*≥0.65 was generated. From these data were calculated ten channel capacities *C* and the averaged alignment fractions *f̅* between misfolded models and original PDB structures with FAST. Figure 6B shows that at *f̅*≈0.6 channel capacity *C* undergoes a sharp increase toward the maximum value at 0.0105 bits per amino acid symbol (with *f̅* = 1 for native structures). This increase thus confirmed that higher channel capacities were indicative of tertiary structures closer to the native state. We note that the total number of contacts did not necessarily indicate higher structural quality: native (*f̅* = 1) structures had 136055 backbone contacts while the misfolded model set with *f̅* = 0.68 had a larger number, 154994.

Random substitutions of symbols in *S_A_* ^*^ represent structural deviations to such extent that the nearest code word in the code book *A* ^*^ is changed, leading to decoding error. Even small variations in geometry can change decoding; for example, from the twenty nuclear magnetic resonance models of human ubiquitin in PDB 1C3T (CATH 3.10.20.90) the carbon backbones of the first and the second model align with RMSD (root mean square deviation) of 0.62 Å. However, in contact vector space both structures are sufficiently divergent to have different nearest neighbor code words: the first decodes to ‘2’ and the second to ‘5’ (Fig. 3). The PDB file 1EO6 also represents two ubiquitin chains (A and B; CATH 3.10.20.90) solved with X-ray crystallography, with chain B having an extra phenylalanine at the C-terminus. Both chains align with a small RMSD of 0.43 Å, but again they are decoded into different code words: ‘4’ for chain A and ‘16’ for chain B.

To further investigate how structural deviations in experimentally determined protein coordinates impair communication, we selected from the original set all *N_X_* = 29945 structures which were solved though X-ray crystallography. Since crystallographic resolution is an indicator of structural quality, i.e., structural models obtained at higher resolution were assumed closer to the native state and thus are less likely to include structural defects, we tested whether *q_e_* ^−^ could discriminate high-resolution from low-resolution structures. Using a filtering procedure, only those structures out of the total *N_X_* were kept which satisfied resolution limits, ranging from 9.50Å to 1.30Å. This yielded thirteen nested sets of structures of increasing crystallographic resolution (Table S2). For each of these sets, channel capacity *C* and sequence-structure fidelity *q_e_* ^−^ were calculated.

**Figure 7:**
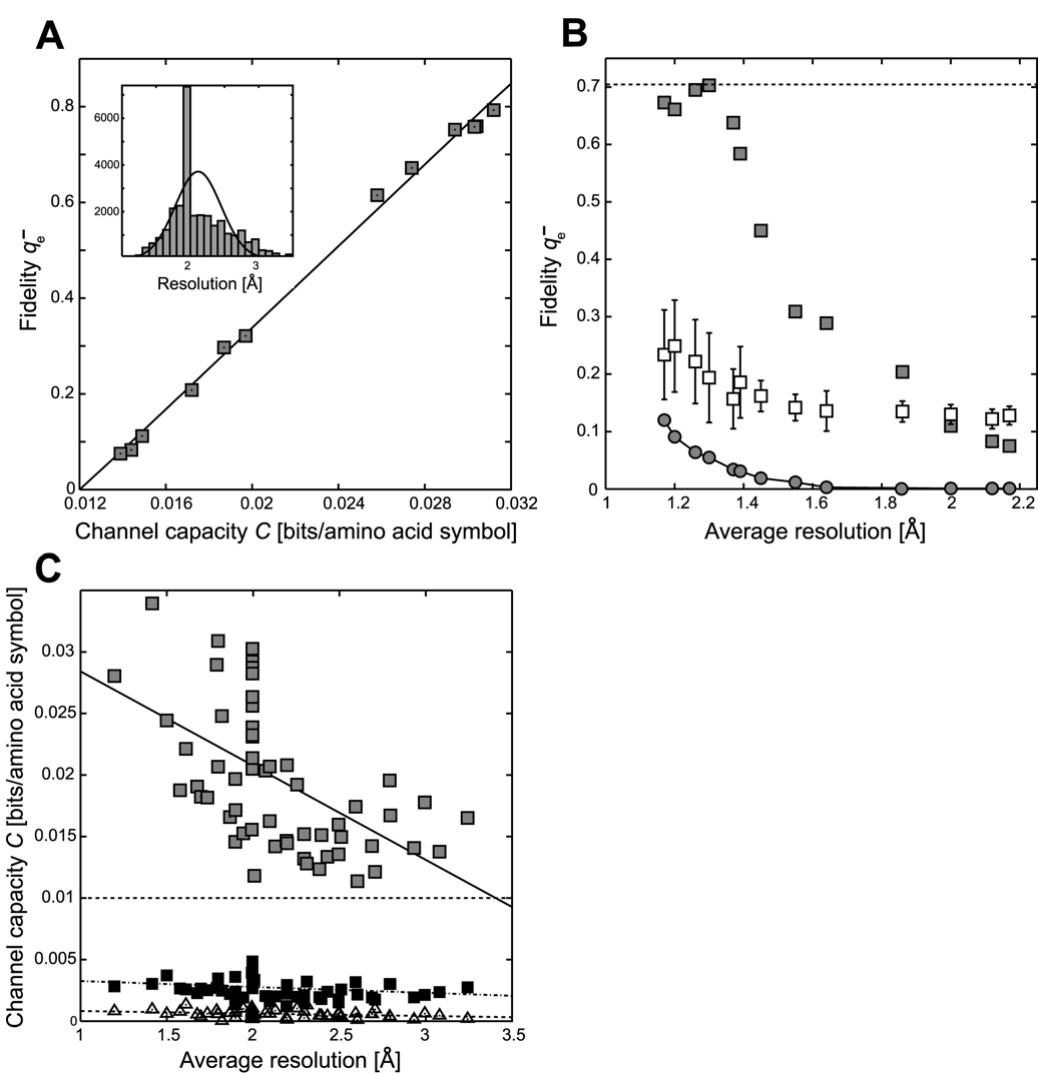
Resolution in crystallographic structures is positively correlated with sequence-structure communication fidelity. (A) Linearity between channel capacity *C* and sequence-structure fidelity *q_e_^−^* for thirteen nested sets of structures with increasing crystallographic resolution (Supporting Information Table S2). Insert shows the distribution of reported crystallographic resolution among 29945 structures. (B) Sequence-structure fidelity as a function of average crystallographic resolution (grey boxes); negative control using thirteen samples of random PDB structures (white boxes); overestimation ΔC for each sample (circles), which were subtracted from the original capacity values. (C) Capacity *C* as a function of average resolution for 59 disjunctive sets of structures ordered by decreasing resolution; each set had a constant number of 500 PDB chains. Negative controls through random permutations in *S_A_* (triangles) and in S_A*_ (filled boxes).

Figure 7A shows a linear relationship between channel capacity *C* and *q_e_* ^−^; linear fitting gave a slope *a* = 42.5 and an offset *b* = −0.51 such that *q_e_* ^−^ = *a C*+*b*, which was consistent with the results in Fig. 5A. The insert in Fig. 7A shows the distribution of reported crystallographic resolutions for all structures; the histogram followed roughly a normal distribution which supported our assumption that resolution was a random source of structural deviations.

Calculations of mutual information from finite statistical samples are systematically overestimated [36], and this positive bias in mutual information had to be considered in our values for *C* and *q_e_* ^−^. Under the assumption |*A*| |*A* ^*^| = 400≪|*S_A_*|, i.e., the number of samples is still much larger than the number of relative frequency bins, the mutual information *C* is overestimated by
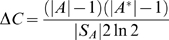
This term, which decreases with larger sample size |*S_A_*|, had to be subtracted from the values of *C*. Due to linearity between *C* and *q_e_^−^* (Fig. 6A), it proportionally reduced the sequence-structure fidelity by the amount Δ*q_e_* ^−^ = *a* Δ*C*. To warrant the above assumption, 400≪|*S_A_*|, our smallest sample contained 91556 amino acid symbols which corresponded to 424 protein chains (Table S2 in Supporting Information).

Figure 7B shows Δ*q_e_* ^−^ (circles) as a function of average crystallographic resolution and the corrected values of sequence-structure fidelity *q_e_* ^−^ (filled boxes). Structural resolution was well correlated with fidelity, ranging from *q_e_* ^−^ = 0.08 at 2.17 Å average resolution to *q_e,max_* ^−^ = 0.71 at 1.30 Å. For average resolutions below ∼1.30 Å fidelity saturated slightly below *q_e,max_* ^−^.

Several controls supported this observation. First, we generated for each of the thirteen sets ten randomly chosen sets of equal size, thus by mixing high-resolution with low-resolution structures. The resulting values *q_e_* ^−^ (open boxes in Fig. 7B) were non-monotonic with increasing resolution and never exceeded an average fidelity of 24%. Second, to cross-validate this maximum fidelity, we added to the sample (Supporting Information, Table S2, 10th entry) random errors in *S_A_* and *S_A_* ^*^ at increasing rates *e_A_*+*e_A_* ^*^, and observed that above *e*_max_≈0.65 the error bound *p_e_* ^−^ approached one (Supporting Information, Fig. S1). This confirmed that *p_e_* ^−^ and *e*_max_ were additive.

In a third control a sampling bias was excluded. Figure 7C shows the relationship between average resolution and channel capacity, this time for successive sets of 500 chains in ordered resolution, which gives a partitioning of the set *N_X_* = 29945 into 59 equal samples (leaving out the last 445 chains in *N_X_*). Although the trend between resolution and channel capacity was noisier, a least square fit identified a negative slope −0.0077±0.0015 bits per amino acid symbol per Å. A negative control using total random permutations in *S_A_* (triangles) and in *S_A_* ^*^ (filled boxes) showed that the signal was well above the random baseline. Together these results supported our hypothesis that both *q_e_* ^−^ and *C* represent sensitive measures of sequence-structure fidelity.

## Discussion

Evidence has been given that protein amino acid sequences and their tertiary structures constitute the source and the destination of a digital communication channel. In direct consequence, Shannon's noisy channel theorem could be applied and a Shannon limit in the sequence-structure map quantitatively predicted.

All relevant Shannon-Weaver communication model components were characterized (source, input, output, decoder, destination) from sequence and structure data except the encoder, i.e., the map from protein sequences (source) onto code blocks (input). A full characterization of the encoding map should explain how an amino acid sequence determines an input contact vector. Although we are not in the position to devise it, there are indicators that such mapping exists. First, the information entropy of output contact vectors, *H_c_* ^*^ = 4.28 bits, is slightly higher than the information entropy of amino acid sequences, *H*(*A*) = 3.90 bits. Thus contact vectors, the inputs and outputs of the channel, retain enough potential information to capture the amino acid code. Second, as amino acid sequences uniquely determine the geometry of the target polypeptides so do contact vectors correspond to unique geometric configurations. This is remarkable because contact vectors, like primary sequences, encode protein structure through a one-dimensional and discrete representation (Supporting Information, Fig. S2).

Without an encoding process, channel capacity and Gallager bound neglected sequence order and were at input sensitive only to errors which changed absolute amino acid sequence composition. Amino acid composition and chain length are important determinants of protein structure [37]–[40], but a correct encoder should be a function of sequence order. In particular since every change in amino acid composition changes amino acid order, but not vice versa, the effect of random errors on channel capacity is underestimated in our analysis. However, the transmission rate, which sets the Shannon limit, is always independent of sequence order.

A possible concern is whether source, block code, and destination are essential components and whether some arbitrary choices could lead to similar results. For example, after replacing the alphabet of twenty amino acid symbols with an alphabet of all 400 dipeptides {AA, AG, …}. In this case the code rate *R*_2_ becomes twice the rate for single amino acids, because *R*_2_≈log_2_ (400)/*n* = 2 log_2_ (20)/*n*≈2*R*. The corresponding capacity *C*_2_ does not change, however, as the chain rule for mutual information *I* gives *C*_2_ = *I*(*A*, *A*; *A* ^*^) = *I*(*A*; *A* ^*^)+*I*(*A*|*A*; *A* ^*^), and the second term is null (consecutive amino acids residues are practically uncorrelated [13]). For our data sets this yields *C* = 0.016 bits/amino acid symbol and *R*_2_ = 0.020 bits/amino acid symbol. Thus, given a block code, Shannon's theorem prohibits an arbitrary increase of the rate by taking blocks of multiple amino acid symbols. This example illustrates that the problem in communication theory is not a choice of alphabets but, critically, the identification of a block code that satisfies Shannon's theorem. Although other block codes may be found, our results demonstrate communication with a block code of contact vectors.

### Shannon limit between sequence and structure

As its key result, the analysis suggests a Shannon limit between protein amino acid sequences and structures at a limit channel capacity of *C* = 0.010 bits per amino acid symbol. This limit, defined at the point where capacity *C* equals channel rate *R*, is a necessary consequence of Shannon-Weaver communication. It is here proposed as an information barrier which needs to be overcome in order to establish communication between sequences and structures. Three main lines of evidence support the Shannon limit hypothesis: first, atomic coordinates from native or close to native structures always lead to capacities higher than *C* = 0.010 (Fig. 4B, 7A–C); second, realistic models of unfolded proteins and random coils yield capacities below this value (Fig. 5D, 6B); and third, random substitutions in primary sequence reduce channel capacity to the limiting point (Fig. 5A).

The Shannon limit thus marks a specific threshold below which communication in sequence-structure ensembles is predicted to cease if errors accumulate above a critical rate. This situation resembles an error catastrophe, i.e., the complete loss of biological information due to excessive noise and errors. However, both concepts should not be confused [41]: the Shannon limit generally follows from errors in digital communication while the term *error catastrophe* originated from a mathematical model of molecular evolution [42].

In their study of protein contact potentials, Cline et al. [12] measured the mutual information of pairwise amino acid residue contacts in 208 protein structures. Using conventional properties of the amino acids they found that only ∼75% of the total 0.04 bits per contact mutual information could be attributed to hydropathy, charge, disulfide bonding, and burial, hence leaving an uncharacterized Δ*I_cp_* = 0.01 bit per contact. We suggest that this extra information represents the Shannon limit at *C* = *R* = 0.010 bits per amino acid symbol. This possibility arises when both numbers, *R* and Δ*I_cp_*, are given the same units by considering *n_c_* = 331, the average number of contacts per contact vector. The Shannon limit then becomes *R*(*n*/*n_c_*) = 1.2×10^−2^ bits per contact, a number that is consistent with Δ*I_cp_*.

### Sequence-structure fidelity

The second result is the identification of a sequence-structure fidelity measure, *q_e_^−^* = 1−*p_e_^−^*, which estimates the probability of correct structural decoding. This fidelity measure decreases with increasing rates of random error in primary sequence and in tertiary structure, and for near atomic resolution structures its value saturates at a maximum of ∼70% (Fig. 7B). This maximum fidelity level corresponds to a capacity of 2.8×10^−2^ bits/amino acid symbol (Fig. 6A), or 3.4×10^−2^ bits/contact, which is in line with previous data on mutual information in protein contact pairs estimated at 0.02 [11] and at 0.04 bits per contact [12]. It is notable that these independent results imply that any communication channel between sequence and structure requires block lengths of at least *n*_min_ = *H*(*A*)/0.04≈100 and *n*_min_≈200, respectively. These are lower bounds consistent with our choice, *n* = 400.

The statistical detection of high-resolution structures with *q_e_* ^−^ appears perhaps questionable, given that an entire polypeptide chain is represented by a single letter in *A* ^*^. However, this result is supported by the fact that (a) above the Shannon limit a single letter carries around 4 bits of information which, together with sequence length, were sufficient to determine the correct fold among single domain structures; and (b) that even small structural variations at atomic resolution are detectable through decoding with contact vectors. Thus once the Shannon limit is overcome, only a few bits of information are necessary to characterize a protein's fold. It is also noted that our analysis requires large enough ensembles of primary and tertiary structures that meet conditions on sampling, |*A*| |*A* ^*^| = 400≪|*S_A_*|, and on entropy balance between source and destination, *H*(*A*)≈*H*(*A* ^*^). Because both conditions are not met for single structures, the fidelity measure *q_e_* ^−^ differs from other computational approaches which often assign a quality measure to a single structure [43], [44].

Since *p_e_* ^−^ estimates the likelihood of decoding error, while reaching a minimum for near-native structures at *p*_e,min_^−^ = 1−*q*_e,max_≈30% (Fig. 6B), it follows that in our model at least ∼30% of all folded polypeptide chains are decoded with error. Is this error estimate biologically relevant? There has been compelling experimental evidence that in eukaryotic cells about ∼30% of all newly synthesized proteins are degraded within minutes of their ribosomal generation [14], [15], [45]. These rapidly degraded ribosomal products are probably not due to short-lived proteins that achieve their native state, but likely constitute misfolded *defective ribosomal products* (DRiPs) which are degraded either by the ubiquitin-proteasome or by a novel and ubiquitylation independent pathway, respectively [46].

If this experimentally determined rate of defective ribosomal products is representative for errors during protein synthesis then it becomes consistent with the maximum fidelity limit on sequence-structure communication derived from our data. This consistency may further suggest that the biological transformation of amino acid sequence into folded protein is an inherently error prone cellular communication process, where many synthesized polypeptides do not make it into native protein structures.

## Supporting Information

Table S1 Table of all 20 structural code words in the code book A* as identified with the *k*-means clustering algorithm among *NP* = 31609 tertiary structures. A *vigesimal* (base-20 numeral system) representation is used for contact vectors by alphabetical ordering of amino acid symbols, {A,C,D,…,Y}. This number representation was convenient because among all *NP* chains only a negligible fraction had contact vector components above 400. It was therefore sufficient to represent contact vectors through an ordered string of values between 0 = (‘Aa’) and 399 = (‘Yy’). For example, a contact vector (*y*_3_ = 320, *y*_4_ = 39; *y*_5_ = 2, *y*_6_ = 0,…, *y*_402_ = 0) is written as ‘TaCyAdAa’, where repetitive zero entries at the end were removed. CM (contact metric) indicates the contact metric distance between a code word and its nearest chain in the PDB along with available CATH structural classification at architecture level.
(0.03 MB PDF)

Table S2 Thirteen nested sets of structures from the Protein Data Bank with increasing crystallographic resolution.
(0.03 MB PDF)

Figure S1 Negative control for Gallager bound by imposing additional random errors. Increase in Gallager error bound due to errors (*eA*+eA*) for the sample of |*SA*| = 204677 and |SA*| = 940 (10th entry in Table S2). Line depicts an exponential least square fit, 1−exp(−*xa*), with *a* = 1.2. Arrow indicates the highest numerical value 0.9994 below one; numerical resolution of the statistical sample was <10^−5^.
(0.40 MB TIF)

Figure S2 Correspondence between the normalized contact metric for contact vectors, *dN*, and maximum alignment RMSD with color encoded alignment coverages for 10,000 random PDB pairs. Small contact metric values, *dN*<0.04, imply geometrical similarity or near identity between two structures.
(1.37 MB TIF)
