# Supplementary material for: Random Amino Acid Mutations and Protein Misfolding Lead to Shannon Limit in Sequence-Structure Communication
Source: PLoS One. 2008 Sep 1;3(9):e3110. doi: 10.1371/journal.pone.0003110 (PMC2518838; doi:10.1371/journal.pone.0003110)
Supplement: Table S2 — Thirteen nested sets of structures from the Protein Data Bank with increasing crystallographic resolution. (0.03 MB PDF) [file pone.0003110.s002.pdf]

Min. resolution [Å]	Average resolution [Å]	Number chains $\|S_{A^*}\|$	Number a.a. symbols $\|S_A\|$
9.50	2.17	29,945	7,558,845
3.00	2.12	28,576	6,939,692
2.50	2.00	23,810	5,769,216
2.00	1.86	15,295	3,428,218
1.85	1.64	6,210	1,404,887
1.75	1.55	4,009	989,032
1.60	1.45	2,321	517,649
1.55	1.39	1,656	364,789
1.50	1.37	1,443	314,645
1.45	1.30	940	204,677
1.40	1.26	787	173,793
1.35	1.20	509	111,194
1.30	1.17	424	91,566
