# Supplementary figures and images for: Random Amino Acid Mutations and Protein Misfolding Lead to Shannon Limit in Sequence-Structure Communication
Source: PLoS One. 2008 Sep 1;3(9):e3110. doi: 10.1371/journal.pone.0003110 (PMC2518838; doi:10.1371/journal.pone.0003110)

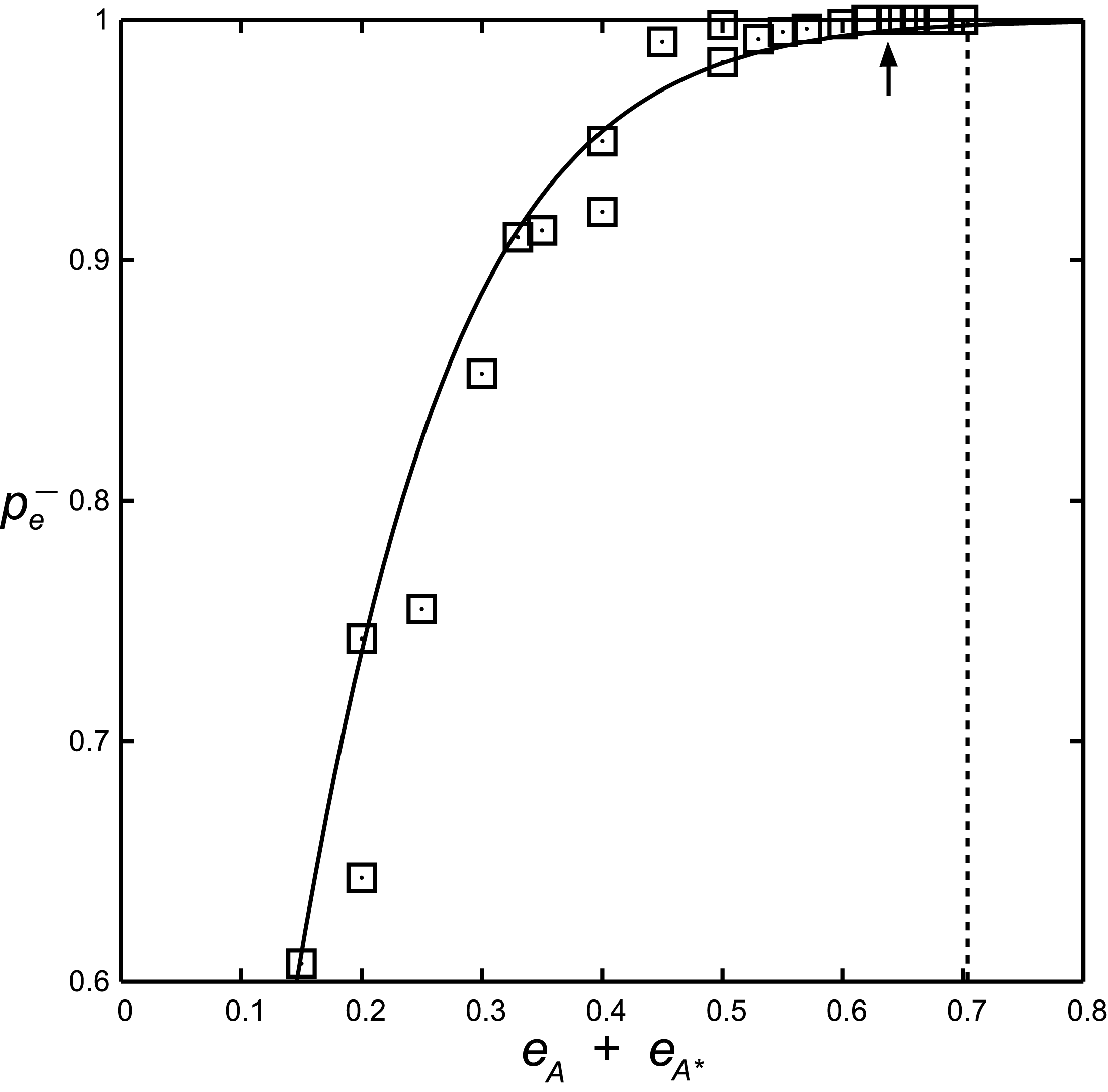

Supplement: Figure S1 — Negative control for Gallager bound by imposing additional random errors. Increase in Gallager error bound due to errors (eA+eA*) for the sample of |SA| = 204677 and |SA*| = 940 (10th entry in Table S2). Line depicts an exponential least square fit, 1−exp(−xa), with a = 1.2. Arrow indicates the highest numerical value 0.9994 below one; numerical resolution of the statistical sample was <10−5. (0.40 MB TIF) [file pone.0003110.s003.tif]

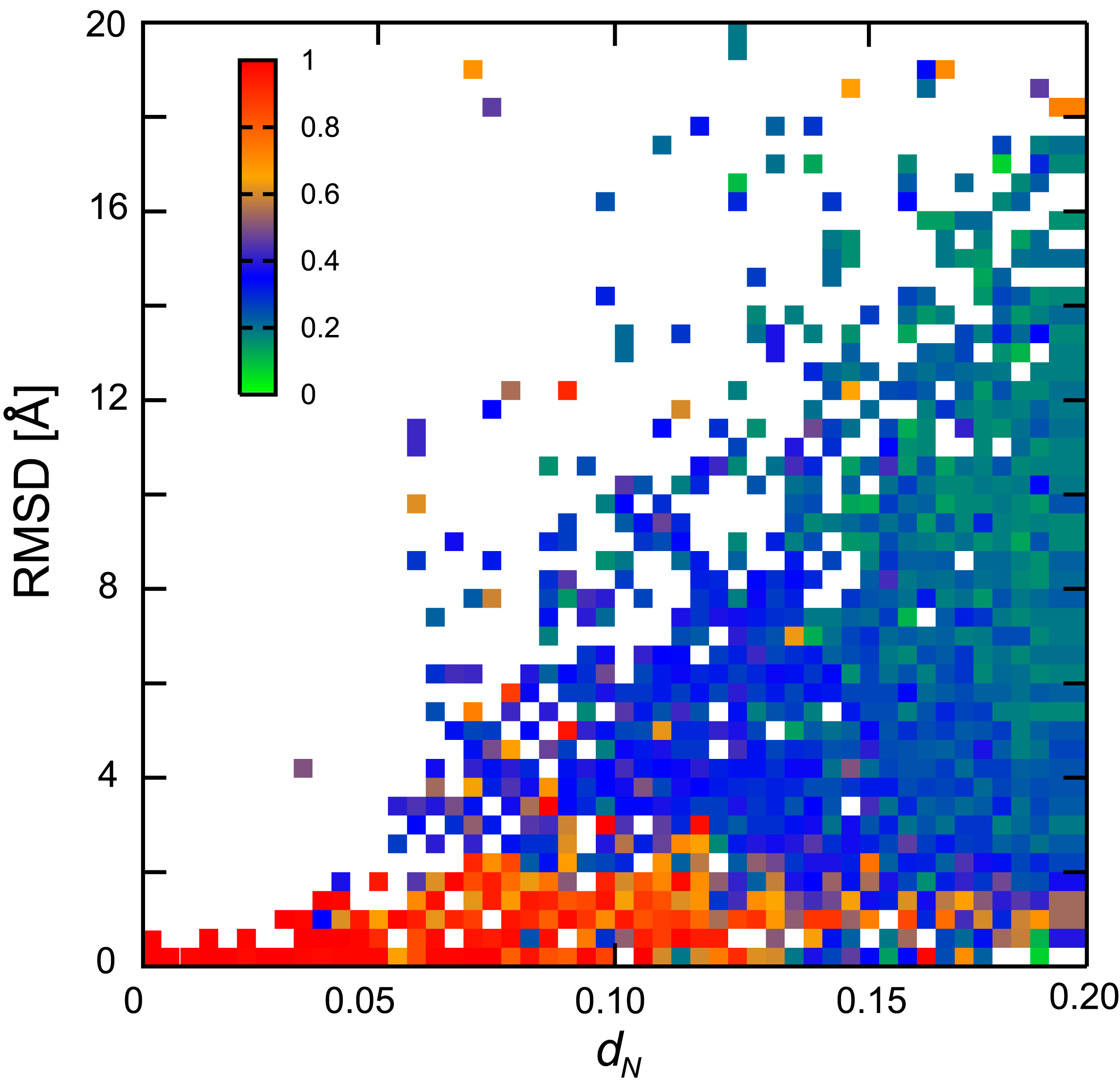

Supplement: Figure S2 — Correspondence between the normalized contact metric for contact vectors, dN, and maximum alignment RMSD with color encoded alignment coverages for 10,000 random PDB pairs. Small contact metric values, dN<0.04, imply geometrical similarity or near identity between two structures. (1.37 MB TIF) [file pone.0003110.s004.tif]
