# Supplementary material for: Random Amino Acid Mutations and Protein Misfolding Lead to Shannon Limit in Sequence-Structure Communication
Source: PLoS One. 2008 Sep 1;3(9):e3110. doi: 10.1371/journal.pone.0003110 (PMC2518838; doi:10.1371/journal.pone.0003110)
Supplement: Table S1 — Table of all 20 structural code words in the code book A* as identified with the k-means clustering algorithm among NP = 31609 tertiary structures. A vigesimal (base-20 numeral system) representation is used for contact vectors by alphabetical ordering of amino acid symbols, {A,C,D,…,Y}. This number representation was convenient because among all NP chains only a negligible fraction had contact vector components above 400. It was therefore sufficient to represent contact vectors through an ordered string of values between 0 = (‘Aa’) and 399 = (‘Yy’). For example, a contact vector (y 3 = 320, y 4 = 39; y 5 = 2, y 6 = 0,…, y 402 = 0) is written as ‘TaCyAdAa’, where repetitive zero entries at the end were removed. CM (contact metric) indicates the contact metric distance between a code word and its nearest chain in the PDB along with available CATH structural classification at architecture level. (0.03 MB PDF) [file pone.0003110.s001.pdf]

Label	Vigesimal contact vector	Nearest PDB chain (CM)	CATH architecture
1	QdMpChAtAkAhAgAfAfAfAfAeAeAeAdAdAdAdAdAdAeAeAdAeAeAeAeAdAd AdAdAdAdAcAcAcAcAcAcAcAcAcAcAcAcAcAcAcAcAcAcAcAcAcAaAcAa	1ia7A (102)	1.50
2	AtAiAdAdAdAdAcAdAdAdAdAdAdAdAcAcAcAcAcAcAcAcAaAaAaAaAaAaAa AaAaAaAaAaAaAaAaAaAaAaAaAaAaAcAcAa	1a7bA (10)	2.60
3	CfApAfAeAdAdAdAdAdAdAdAdAdAdAdAdAdAdAdAcAcAcAcAcAcAcAa	1wepA (19)	3.30
4	DaCmAfAeAdAcAcAcAcAcAcAcAcAcAaAcAa	1mhlA (11)	4.10
5	FkDcAqApAmApAmAnAmAmAmAmAmAlAmAlAlAiAiAhAhAhAgAfAfAeAeAeAeAe AdAdAdAdAdAcAcAdAcAcAdAcAcAcAcAcAcAcAcAcAcAcAaAcAaAaAcAcAc AcAcAcAcAcAcAcAcAaAaAaAaAaAaAaAaAaAaAaAaAcAaAaAaAaAcAcAaAcAa	1z0hA (125)	1.10, 3.30
6	GcDeAhAgAeAeAdAdAdAdAcAcAcAcAcAcAeAhAtCeDaCtCiArAqAqAwAqAlAg AkAgAfAeAfAhAgAgAfAfAdAaAcAcAcAeAcAaAaAaAaAaAaAaAaAaAaAaAaAa AaAaAaAaAaAaAaAaAaAaAcAcAaAaAaAaAaAaAaAaAcAcAa	1gwbB (207)	3.80
7	DpCyAhAfAdAdAcAcAcAcAcAcAcAcAcAcAcAa	1egwA (13)	1.10
8	FcEcAkAgAeAeAdAdAdAdAdAdAcAcAcAcAcAcAcAcAcAcAcAcAcAcAcAcAcAc AcAcAcAcAcAcAcAcAaAcAcAaAaAaAaAaAaAcAcAa	1t9iA (34)	3.10
9	ImGwAqAlAgAeAdAdAdAdAdAdAdAdAcAcAcAdAcAdAdAdAeAeAdAdAdAdAcAc AcAcAcAcAcAcAcAcAcAcAcAcAcAcAa	1qhhB (64)	3.40
10	EeCsAhAgAfAfAeAfAfAeAeAeAfAfAgAfAfAeAfAeAeAeAdAdAcAcAdAdAcAc AdAcAdAcAcAcAcAaAaAcAaAaAaAcAcAdAdAdAcAcAcAaAcAaAaAaAaAaAaAc AcAcAaAcAcAaAaAaAaAaAaAaAcAaAaAaAaAaAaAcAcAaAaAaAaAaAaAaAcAa	1l3aC (63)	2.30
11	EhDpAhAfAdAdAcAcAcAcAcAcAcAcAcAa	1etoA (13)	1.10
12	AlAgAdAcAa	1fu2B (1)	n.a.
13	YyWaDaCdAqAlAkAiAhAhAgAfAfAfAfAfAeAfAeAeAeAeAeAeAeAeAdAdAdAd AdAdAdAdAdAeAdAeAdAdAdAdAdAdAdAcAdAcAcAcAcAcAcAcAcAcAcAcAcAc AaAaAaAcAcAaAaAaAcAaAcAaAaAaAcAcAcAaAcAaAaAaAaAcAa	1is2A (145)	1.10, 2.40, 1.20
14	ClCcAeAdAcAcAcAcAcAcAcAcAa	1jsuC (7)	4.10
15	GyGaAmAhAeAdAcAcAcAcAcAcAcAcAaAaAcAcAcAcAcAcAcAcAcAcAaAaAc AcAa	1e7lA (29)	n.a.
16	DaAwAhAhAgAgAfAfAfAfAfAgAfAgAfAfAfAfAeAeAdAdAdAcAcAcAcAcAcAc AcAcAcAcAcAcAcAcAcAcAcAcAcAcAcAcAcAcAcAcAcAcAcAcAcAcAcAcAcAa AaAaAaAaAaAaAaAaAaAaAaAaAaAaAaAaAaAaAaAaAaAaAaAaAaAaAaAaAaAa AaAcAa	1hpwA (55)	3.30
17	GlFcAnAkAgAfAeAeAeAeAeAeAdAdAdAdAdAdAdAdAdAdAdAdAdAcAcAcAc AcAcAcAcAcAcAcAcAcAcAcAcAcAcAcAcAaAcAaAcAa	1xmoD (55)	1.10, 3.10
18	IwFgCeCaArAvAsAsArArArApAqArAsAsAtAmAmAlAkAkAlAkAiAhAgAgAgAf AeAeAeAeAeAeAdAeAdAdAdAdAdAdAdAdAdAdAcAcAcAcAcAcAcAcAcAcAcAc AcAcAcAaAcAcAcAaAaAaAaAaAaAaAaAaAaAaAaAaAaAaAcAa	1hcmA (159)	1.10, 2.140
19	FqEvAiAfAdAdAcAcAcAcAcAcAcAaAaAaAaAaAaAaAaAcAaAaAaAcAcAaAa AaAaAaAaAcAa	1q05A (18)	1.10
20	LnIqAvApAhAfAeAeAeAeAdAdAdAdAdAdAdAdAdAdAdAdAdAdAdAdAdAdAdAd AdAdAcAcAcAcAcAcAcAcAcAcAcAcAcAcAcAcAaAcAa	1mq2A (76)	1.10, 3.30
